# A High-Sensitivity Flexible Eddy Current Array Sensor for Crack Monitoring of Welded Structures under Varying Environment

**DOI:** 10.3390/s18061780

**Published:** 2018-06-01

**Authors:** Tao Chen, Yuting He, Jinqiang Du

**Affiliations:** Aeronautic Engineering College, Air Force Engineering University, 1 Baling Road, Xi’an 710038, China; heyuting1966@gmail.com (Y.H.); dujinqiang1983@gmail.com (J.D.)

**Keywords:** flexible eddy current array sensor, structural health monitoring, sensitivity, crack detection, effects of stress and temperature variations

## Abstract

This paper develops a high-sensitivity flexible eddy current array (HS-FECA) sensor for crack monitoring of welded structures under varying environment. Firstly, effects of stress, temperature and crack on output signals of the traditional flexible eddy current array (FECA) sensor were investigated by experiments that show both stress and temperature have great influences on the crack monitoring performance of the sensor. A 3-D finite element model was established using Comsol AC/DC module to analyze the perturbation effects of crack on eddy currents and output signals of the sensor, which showed perturbation effect of cracks on eddy currents is reduced by the current loop when crack propagates. Then, the HS-FECA sensor was proposed to boost the sensitivity to cracks. Simulation results show that perturbation effect of cracks on eddy currents excited by the HS-FECA sensor gradually grows stronger when the crack propagates, resulting in much higher sensitivity to cracks. Experimental result further shows that the sensitivity of the new sensor is at least 19 times that of the original one. In addition, both stress and temperature variations have little effect on signals of the new sensor.

## 1. Introduction

Crack damage has become the main factor to the safety of structures, and connection parts of metal structures are generally stress-concentration areas where cracks are most likely to occur. As the most important structural connection in various equipment and facilities, welds are also vulnerable to cracks. Many equipment and facilities, such as cranes, power generation equipment, pressure vessels, pipelines, bridges, ships, etc., have all experienced catastrophic accidents due to the fracture of welded structures. According to a statistic of pressure vessels, more than 70% of accidents are caused by cracks [1]. In the inspection statistics for many harbor cranes, the potential threat to the safe use of crane metal structures is mainly cracks at weld seams, and the resulting failures account for approximately 80% of all failures [2]. Despite all kinds of cracks in these cranes, these equipment are still in service due to various reasons, which brings huge safety risks.

Structural health monitoring (SHM) technology can promptly detect defects and improve the safety and maintainability of equipment and facilities. Current SHM technologies include optical fiber [3,4,5,6], CVM sensor [7], acoustic emission [8,9], ultrasonic [10,11], and other sensor technologies [12,13]. Flexible eddy current sensors are manufactured by the flexible printed circuit technology, which can be used for crack monitoring of various curved structures. Alexi Rakow and Fukuo Chang [14] developed a flexible eddy current sensor integrated with the bolt for monitoring crack growth of structures around bolt holes. The signals of the sensor are affected by stress changes during the experiment, resulting in a lower sensitivity to cracks. JENTEK Sensors [15,16,17,18,19] has developed a variety of Meandering Winding Magnetometer (MWM) sensors for damage detection and quality evaluation of metal structures, including aeroengine blades, turbine disks, pressure vessels, oil pipelines and other structures with complex curved surfaces. In addition, the sensor has been used to crack monitoring of bolt-jointed structures. However, the influence of the service environment on the sensor output signal has not been studied. In a previous paper [20], the influence of temperature changes on signals of a rosette eddy current sensor is studied. Results show that the ambient temperature has a great influence on the output signal of the sensor by affecting the conductivity and permeability of the structures under testing.

Therefore, the influence of stress, temperature, and crack on the output signal of a traditional FECA sensor for crack monitoring of welded structures was studied. A finite element model was established to study the effect of crack perturbation on eddy currents distribution and output signals. According to the results obtained by the finite element analysis, the HS-FECA sensor was proposed. The sensitivity of the sensor to cracks was studied using finite element simulation and experiments.

## 2. Experiments of Traditional FECA Sensor

### 2.1. FECA Sensor

**Figure 1:**
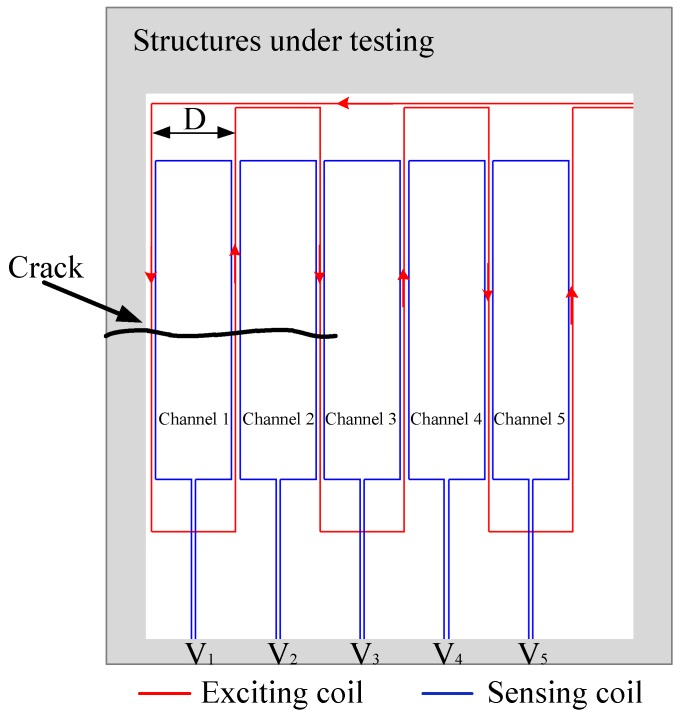
The schematic of a traditional FECA sensor.

The schematic of a traditional FECA sensor is shown in Figure 1. The meandering exciting coil forms several rectangular loops. Each sensing coil lies in the middle of each rectangular loop. When the sensor is working, a time-varying current *I* is applied to the exciting coil to generate eddy currents on the surface of the structure under monitoring, and the varying eddy currents causes each sensing coil to generate an induced voltage *V*. When the crack propagates below the corresponding sensing channel, the eddy currents of the structure will be changed, resulting in variations of the induced voltage. This paper defines the sensor output signal *A_R_* as:

(1) $$A_{R}=\frac{V}{I}$$

where *V* is the voltage magnitude of a sensing channel and *I* is the magnitude of the exciting current.

The FECA sensor is manufactured using a flexible printed circuit board manufacturing process, which is comfortable for various complex curved structures. The dangerous part of the welded structure is often located at the weld seam. The FECA sensor can be used for crack monitoring at this curved and rough location.

### 2.2. Effects of Stress Variations on the Output Signal of the Sensor

**Figure 2:**
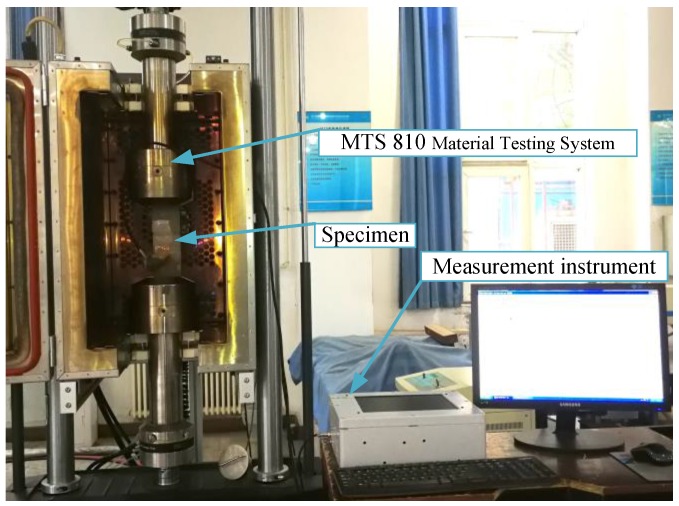
The experimental system for effects of stress and cracks.

**Figure 3:**
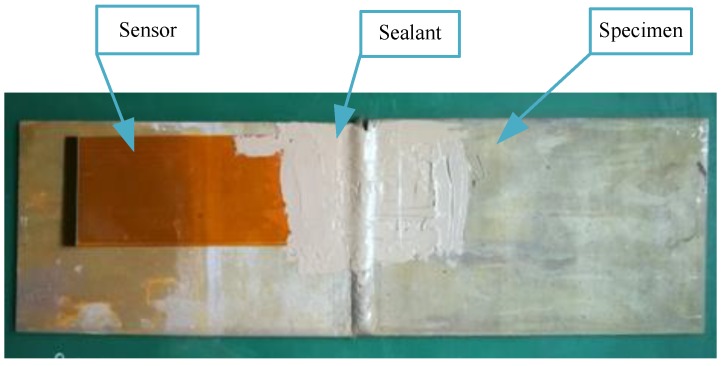
The specimen integrated with the sensor.

The experimental system used is shown in Figure 2. The system includes a measurement instrument, MTS810 material testing system, and the specimen integrated with the sensor (Figure 3). The measurement instrument includes an embedded computer, a stimulation source, a conditioning module and an AD converter. The frequency of the exciting current is 1 MHz in this paper, the sampling rate is 50 MHz and the resolution is 16 bit. The specimen is welded by a single-side welding and double-side forming process. The sensor is directly mounted on the specimen by sealants without any other surface treatment and the sense channels cover the welding seam.

**Figure 4:**
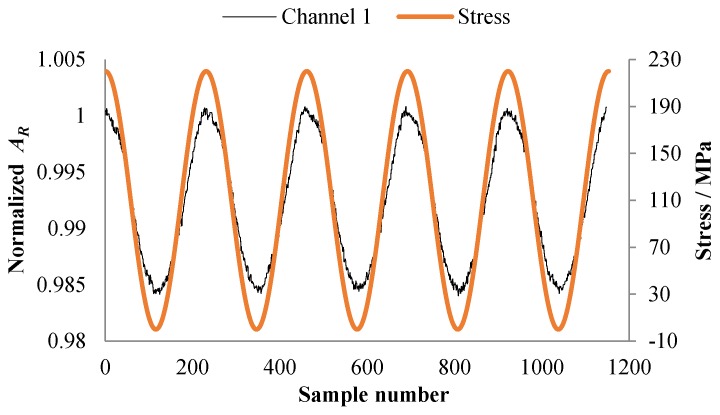
The output signal varies with stress.

Due to the magnetoelastic effect of the ferromagnetic material, output signals of the sensor are affected by the stress. Therefore, before carrying out crack monitoring experiments, the influence of stress on the output signal of the sensor was studied. During the test, a constant–amplitude spectrum (maximum stress *S_max_* = 220 MPa, stress ratio *R* = 0, loading frequency *f* = 0.02 Hz) was applied to the specimen, while signals of the sensor were collected. As shown in Figure 4, the output signal of channel 1 changes with the change of stress, and the varying amplitude is about 1.5%. This would reduce the sensor’s sensitivity to cracks and have a certain impact on crack monitoring.

### 2.3. Experimental Research on Crack Monitoring of Welded Structures

**Figure 5:**
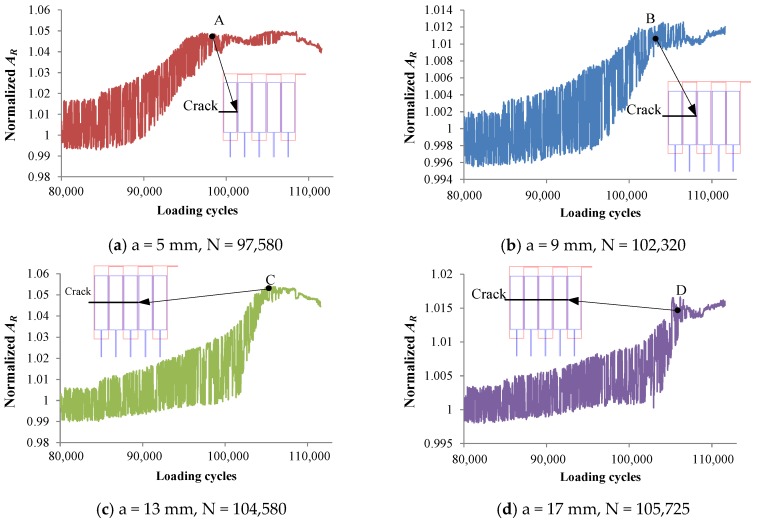

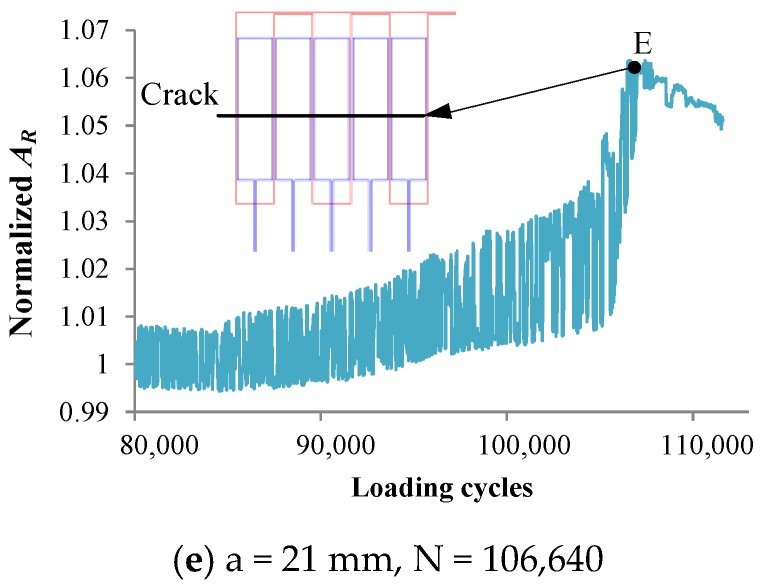
Output signals vary with the growth of crack.

**Figure 6:**
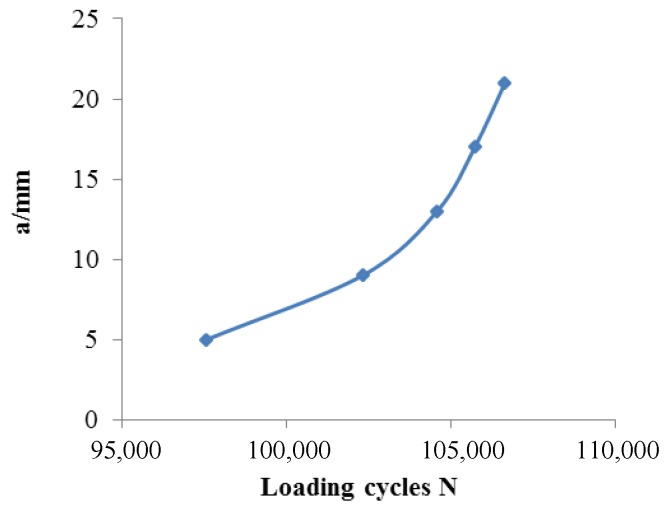
The relation between the crack length and loading cycles.

To perform the crack monitoring experiment, a constant–amplitude spectrum (maximum stress *S_max_* = 220 MPa, stress ratio *R* = 0, loading frequency *f* = 15Hz) was applied to the specimen. As shown in Figure 5, output signal of each channel arrives at each turning point A–E when crack tip propagates to corresponding exciting coil of each channel. Therefore, the loading cycles separately corresponding to crack length a of 5 mm, 9 mm, 13 mm, 17 mm, and 21 mm can be obtained, as shown in Figure 6. It should be noted that because the sensor can be easily affected by stress and the sensitivity of the sensor to cracks is so low, the estimation errors are larger. The maximum change in the output signal of channel 1, channel 3, and channel 5 is approximately 5%, while the maximum change in the output signal of channel 2 and channel 4 is just approximately 1.5%.

In addition, it can be seen in the figure that, when no crack exits in the structure, the output signal of the sensor fluctuates greatly under the effect of stress. When the crack propagates to the corresponding sensing channel, the fluctuation amplitude of the signal increases first and then decreases. This is mainly due to stress concentration in the crack tip region during crack propagation, and there only exists very small stress in the region between the fatigue source and the crack tip.

### 2.4. Effects of Temperature Variations on Output Signals of the Sensor

Both stress and temperature have influences on the electrical conductivity and permeability of the material, causing interference with output signals of the sensor. As shown in previous sections, the change of stress has a greater impact on the output signal of the sensor, but it will not cause excessive interference to the monitoring process. Therefore, this section focuses on the effect of temperature changes on output signals of the sensor.

**Figure 7:**
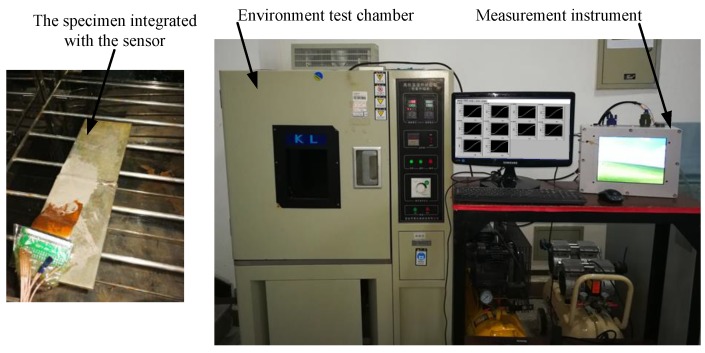
Experimental research on effects of temperature.

**Figure 8:**
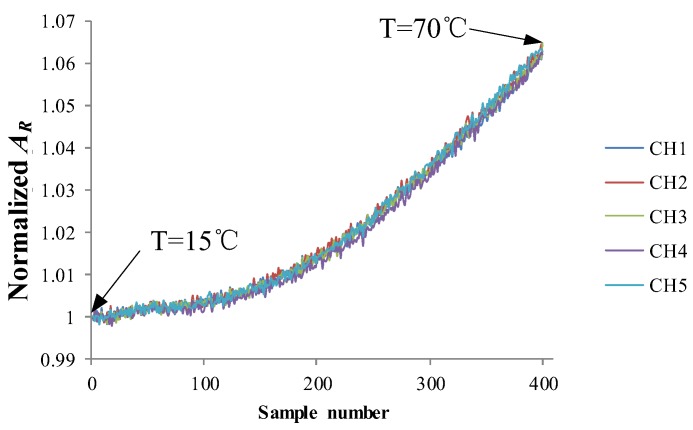
Signals of the sensor when temperature changes.

As shown in Figure 7, during the experiment, the specimen integrated with the sensor was placed in the environmental test chamber. As shown in Figure 8, the sensor’s output signal increases by about 6% in the whole temperature variation process. When the crack grows, variations of output signals are less than 5%. Therefore, changes in the ambient temperature would cause significant interference with output signal of the sensor. Therefore, the sensitivity of the sensor needs to be improved by optimizing the design of the sensor.

## 3. The Finite Element Analyses of the Sensor

**Figure 9:**
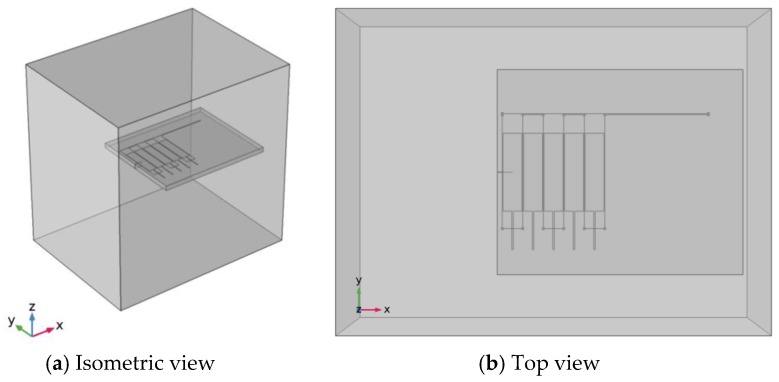
3-D FE model of the sensor.

In this paper, a 3-D finite element model is established in Comsol software [21], as shown in Figure 9. The coil thickness is so small and the exciting current frequency is so high that the skin effect of eddy current in this model is obvious. Therefore, this paper uses the line model to simulate exciting coils and the area model to simulate sense channels. To simulate the crack propagation, a 0.02 mm wide crack is established with a length from 0 mm to 23 mm. The distance between the left most exciting coil and the left edge of the structure is 1 mm. The induced voltage of each sensing channel can be obtained using Formula (2), and then the output signal *A_R_* of each channel can be obtained by Formula (1).

(2) $$V=\iint \frac{dB}{dt}dS$$

where *B* is magnitude of magnetic flux density and *S* is the area of the sense element.

The variation rate of the output signal is defined as:

(3) $$S_{C}=\frac{\Delta A_{R}}{A_{R0}}=\frac{\left|A_{R}-A_{R0}\right|}{A_{R0}}$$

where *S_C_* is the variation rate of the output signal and *A_R_*_0_ is the initial output signal of the sensor.

**Figure 10:**
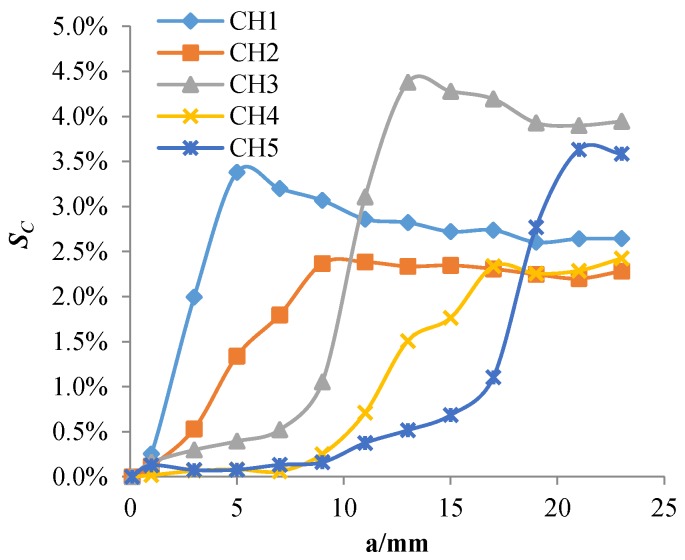
The *S_C_* of each channel varies with the crack growth.

As shown in Figure 10, when the crack propagates to the front-end exciting coil of a channel, the *S_C_* of the channel begins to increase rapidly; when the crack tip just reaches the rear-end exciting coil of the channel, the *S_C_* reaches its maximum value and then slowly decreases. Therefore, the maximum value of *S_C_* can characterize the sensor’s sensitivity to cracks. The simulation results show that the maximum value of the *S_C_* in each channel does not exceed 5% when the crack propagates. The change is so small that the sensor’s sensitivity to cracks is too low.

**Figure 11:**
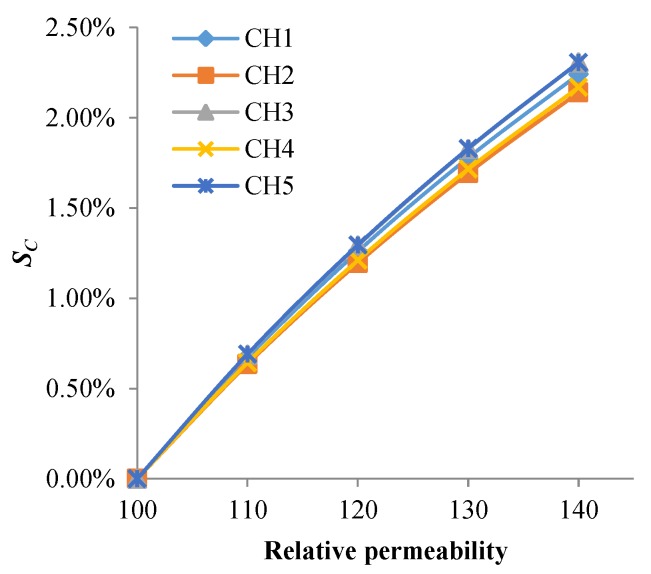
The *S_C_* of each channel varies with permeability of the structure.

**Figure 12:**
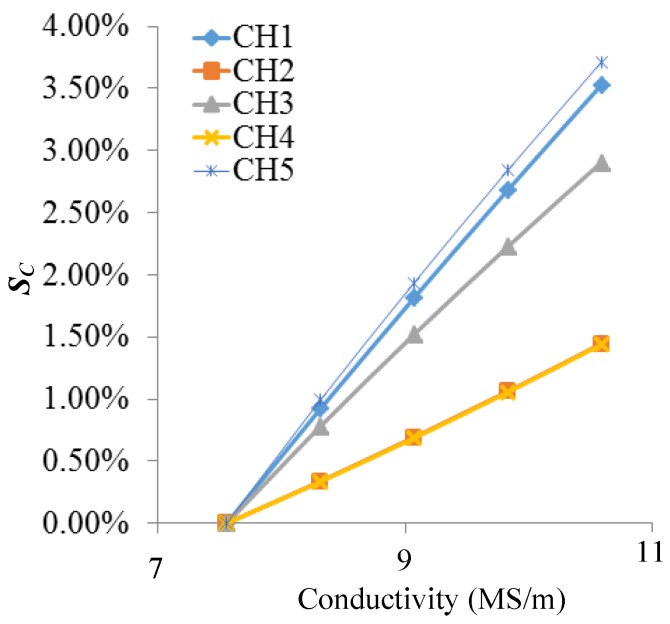
The *S_C_* of each channel varies with conductivity of the structure.

As shown in Figure 11 and Figure 12, changes of permeability and conductivity have a significant effect on the output signal of the sensor. Temperature and stress variations can cause changes of the material’s conductivity and permeability, which is the reason the output signal of the sensor changes with temperature and stress variations.

**Figure 13:**
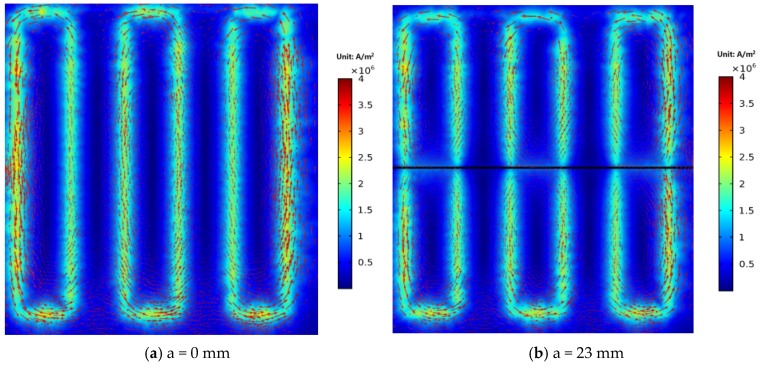
Distributions of eddy currents before and after the crack propagates through the monitoring area.

As shown in Figure 13a, before the crack initiates, three large eddy current loops are induced by exciting coils on the structure surface due to the opposite current flow of adjacent exciting coils, corresponding to channel 1, channel 3 and channel 5, respectively. When the crack propagates through the monitoring area, the three larger eddy current loops are split into six eddy current loops.

**Figure 14:**
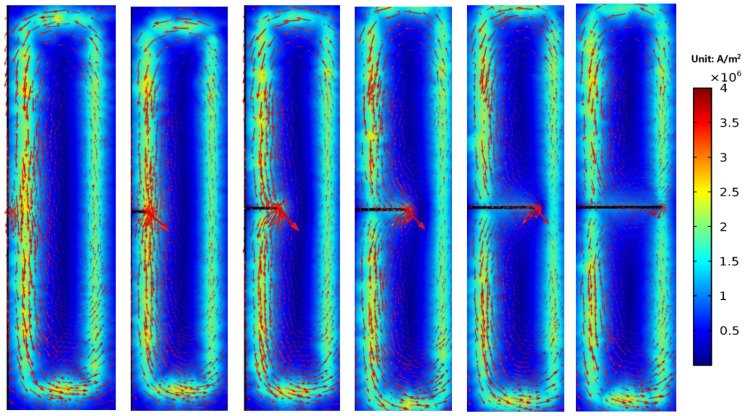
Distributions of eddy currents when the crack propagates from 0 mm to 5 mm.

As shown in Figure 14, when the crack propagates to the front end of channel 1, eddy currents around the crack flows around the crack tip. The crack disturbs the distribution of eddy currents, and *S_C_* of the channel 1 starts to increase. As the crack grows, more and more eddy currents are disturbed, and the *S_C_* continues to increase. When the crack propagates to the middle of the channel 1 (a = 3 mm), it is obvious that part of eddy currents flow toward eddy currents excited by the rear-end coil of channel 1 and form a loop. With the growth of the crack, eddy currents flowing around the crack tip are getting less, and the perturbation effect of the crack tip on eddy currents are also getting weaker. This leads to the fact that the increasing rate of *S_C_* is getting smaller as the crack grows.

**Figure 15:**
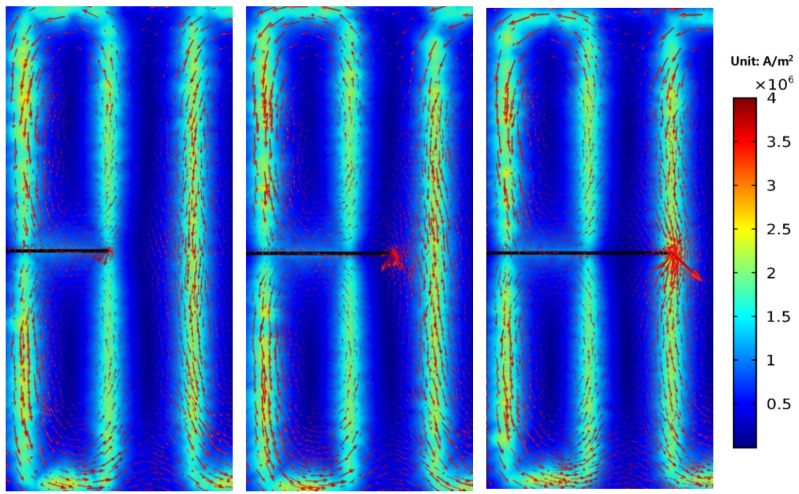
Distributions of eddy currents when the crack propagates from 5 mm to 9 mm.

Figure 15 shows the eddy current distribution when the crack tip propagates in the corresponding area of channel 2. Because directions of exciting currents at the upper end of channel 2 are opposite and two exciting coils are so close, eddy currents in the region do not form a loop similar to that of the channel 1, and the eddy current density in the region is smaller. When the crack propagates in this region, eddy currents flowing around the crack tip are less, resulting in a weaker perturbation effect on eddy currents. Therefore, as shown in Figure 10, the maximum values of *S_C_* for channel 2 and channel 4 are only about 2.4%, which is less than that of channel 1, channel 3, and channel 5 (3.2%, 4.3%, and 4.2%, respectively). This phenomenon is verified in the crack monitoring experiment (Figure 5): the maximum values of *S_C_* for channel 1, channel 3, and channel 5 are all about 5%, while the maximum values of *S_C_* for channel 2 and channel 4 are approximately 2%.

**Figure 16:**
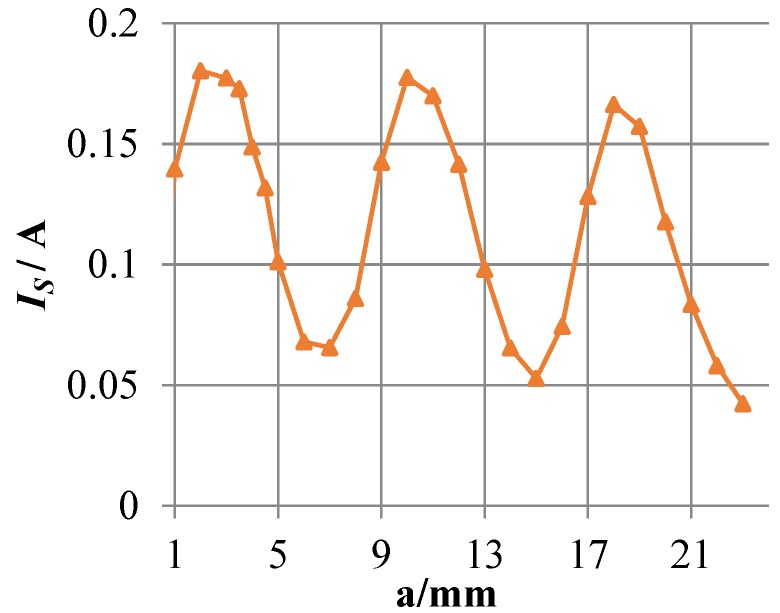
The current magnitude at the crack tip surface *I_S_* varies with the crack growth.

To analyze the perturbation effect of cracks on eddy currents, the current magnitude *I_S_* at the crack tip surface is calculated as the crack propagation, as shown in Figure 16. When the crack propagates in the corresponding area of channel 1, *I_S_* first increases and then decreases. The increase is due to the presence of the crack that cause eddy currents to flow around the crack tip. As the crack propagates, some of the front-end eddy currents start flowing towards the rear-end eddy currents and form a loop, and part of eddy currents continue flowing around the crack tip. In the meantime, *I_S_* begins to decrease, causing the perturbation of the crack tip to eddy currents to begin to decrease. When the crack propagates in the area corresponding to channel 2 (channel 4), *I_S_* first decreases and then increases. This is mainly because the regions corresponding to channels 1, 3 and 5 form a relatively large current loop. Thus, the eddy current density of the corresponding regions of channel 2 and channel 4 is smaller, and the eddy current density at the middle of the two channels is the smallest.

Therefore, adjacent exciting currents in the opposite direction forms current loops when the crack propagates, making eddy currents at the crack tip decrease, reducing the perturbation of the crack on eddy currents, resulting in low sensitivity to crack detection.

## 4. Design of the HS-FECA Sensor

**Figure 17:**
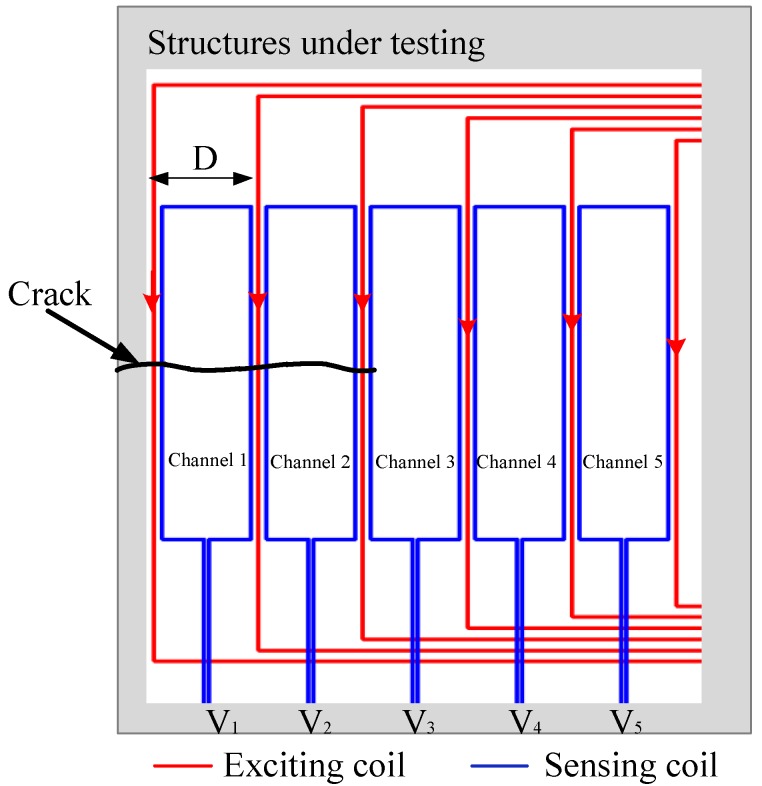
The schematic of the HS-FECA sensor.

As shown in the previous section, the perturbation of the crack tip to the eddy currents plays a major role in the sensitivity to cracks. To improve the sensitivity of the sensor to cracks, this paper proposes a new winding method in which the exciting currents are arranged in the same direction (as shown in Figure 17), so that the channels do not form any eddy current loop when the crack propagates. To verify the validity of this design method, a finite element model is established. In addition to the sensor winding method, the rest of the parameters are the same as those shown in Figure 9.

**Figure 18:**
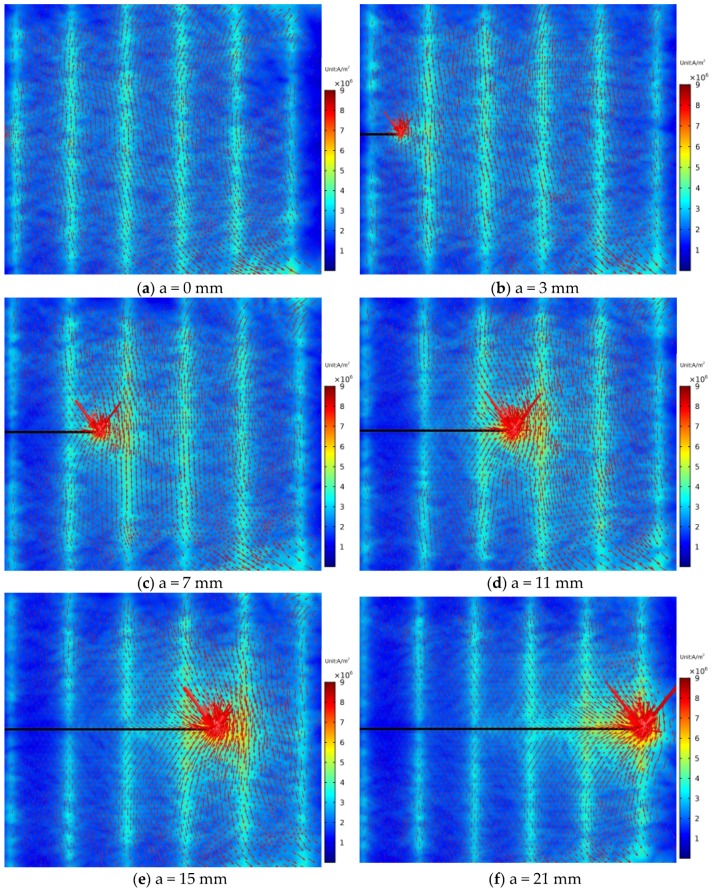
The eddy current distribution in the monitoring region.

**Figure 19:**
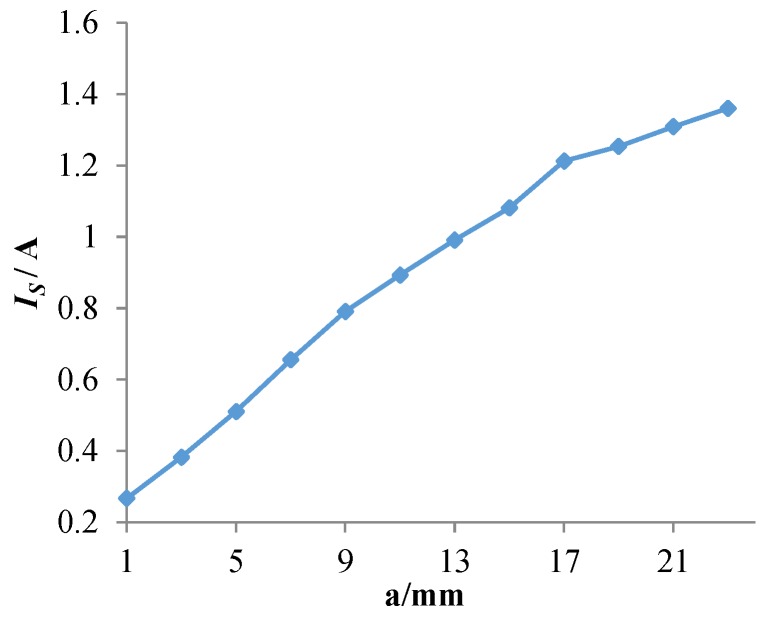
The current magnitude at the crack tip surface *I_S_* varies with the crack growth.

**Figure 20:**
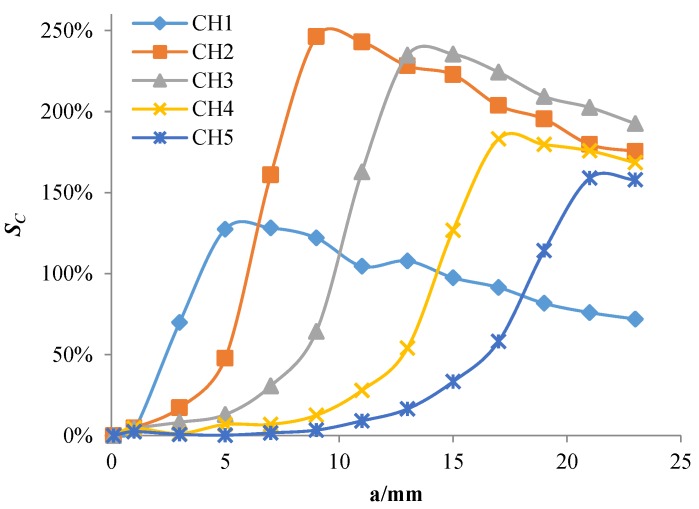
Output signals of the new sensor when the crack propagates.

Figure 18 shows the eddy current distribution in the monitoring area during crack propagation. As shown in the figure, the presence of a crack alters the flowing trajectory of eddy currents near the crack. Because directions of exciting currents are the same, eddy currents also flow in the same direction. Eddy currents near the crack do not form a loop, but always flow around the crack tip. As the crack propagates, more and more eddy currents flow around the crack tip. As shown in Figure 19, the current magnitude at the crack tip surface *I_S_* increases rapidly with crack propagation, which means the perturbation of the crack becomes stronger and stronger. As shown in Figure 20, *S_C_* of each channel in the new sensor has reached more than 120%. Compared to 5% of the traditional sensor, the sensitivity of the new sensor is at least 24 times that of the traditional one.

**Figure 21:**
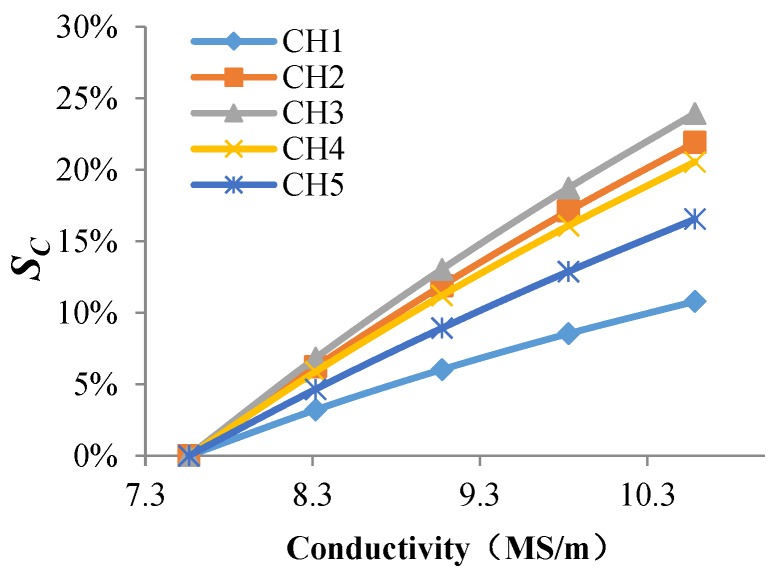
The *S_C_* of the new sensor varies with conductivity of the structure.

**Figure 22:**
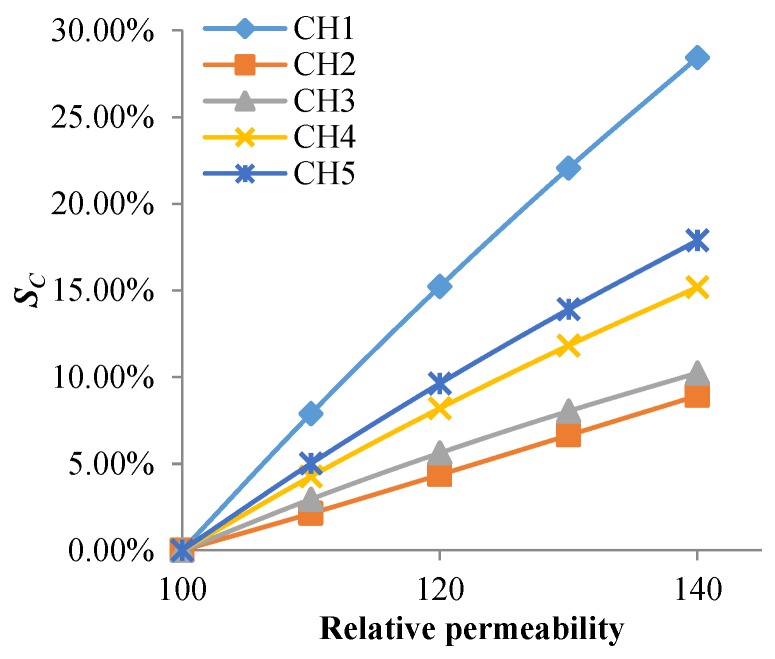
The *S_C_* of the new sensor varies with permeability of the structure.

Figure 21 and Figure 22 show the influence of changes in conductivity and permeability on output signals of the new sensor. Compared with effects of cracks on the output signal, effects of changes in conductivity and permeability are essentially negligible.

## 5. Experimental Verification of the HS-FECA Sensor

**Figure 23:**
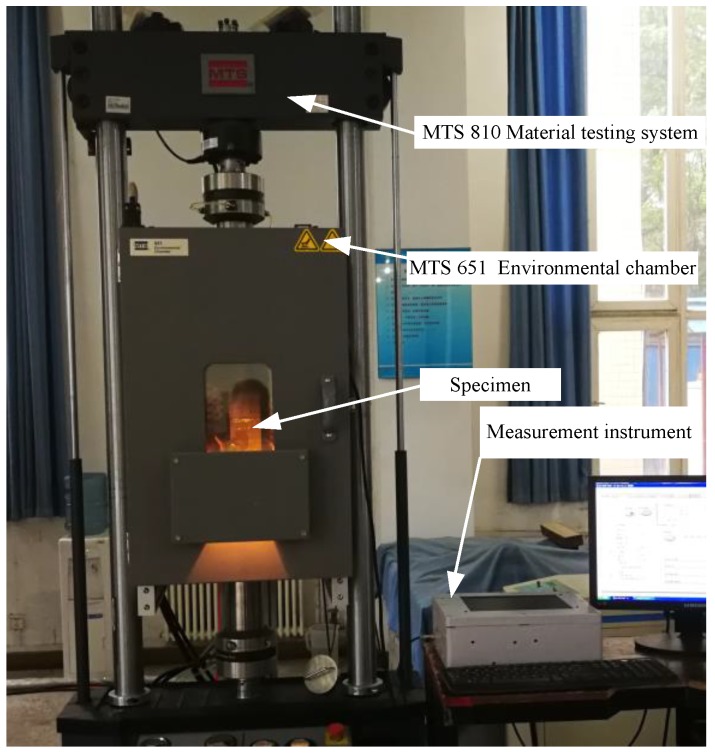
Experimental verification of the HS-FECA sensor.

As shown in Figure 23, to verify the monitoring sensitivity of the HS-FECA sensor, experimental investigations on effects of stress, temperature and crack are performed in this section. The specimen is the same welded structure shown in Figure 3.

**Figure 24:**
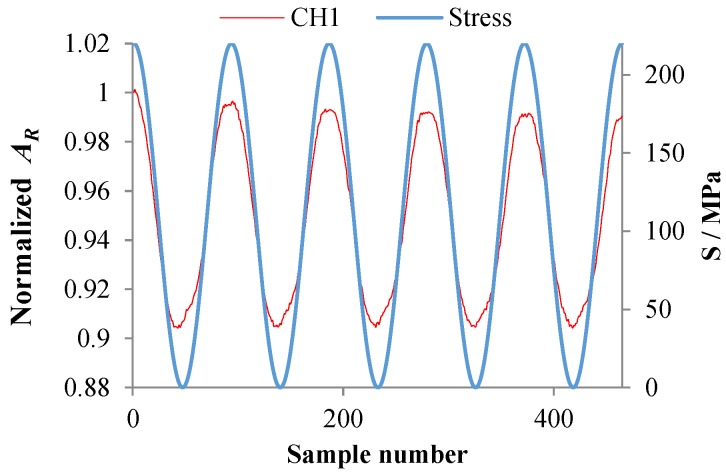
Output signal of the HS-FECA sensor varies with stress.

Firstly, effects of stress on output signals of the HS-FECA sensor were studied. A constant–amplitude spectrum (maximum stress *S_max_* = 220 MPa, stress ratio *R* = 0, loading frequency *f* = 0.02 Hz) was applied to the specimen, while signals of were collected. As shown in Figure 24, the output signal of channel 1 changes with the change of stress, and the varying amplitude of the output signal is about 10%.

**Figure 25:**
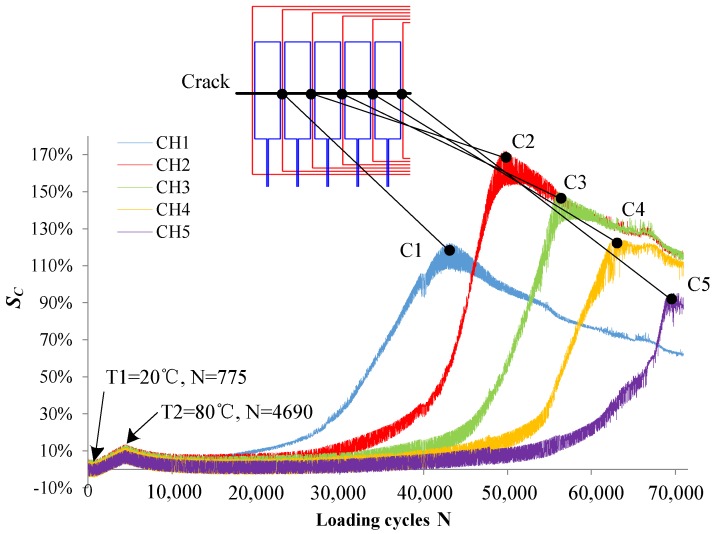
*S_C_* of the HS-FECA sensor varies with loading cycles.

Then, the experiment of crack monitoring under temperature interference is carried out. A constant–amplitude spectrum (maximum stress *S_max_* = 220 MPa, stress ratio *R* = 0, loading frequency *f* = 15 Hz) was applied to the specimen. As shown in Figure 25, when the loading cycles N = 755, the temperature starts to rise from 20 °C to 80 °C. Then, the temperature decreases to the room temperature. When the temperature reaches 80 °C, the maximum *S_C_* of each channel is 8%, 13%, 13%, 12%, and 10%, respectively.

When the crack tip propagates to the rear end of each channel, the signal *S_C_* of each channel reaches the corresponding inflection point (C1–C5). Accordingly, loading cycles N corresponding to crack lengths of 5 mm, 9 mm, 13 mm, 17 mm, and 21 mm can be obtained as 43,064, 49,735, 55,920, 62,669, and 68,777, respectively. Besides, the maximum *S_C_* of each channel is 122%, 172%, 150%, 125%, and 95%, respectively. The sensitivity of the HS-FECA sensor is at least 19 times that of the original sensor. Compared with the variation of output signals resulting from the crack, the effect of temperature and stress on output signals of the sensor is so small that whether there is a crack below the sensor under the actual service environment can be easily judged.

When the sensor is applied to monitor cracks of actual welded structures, the channel numbers of the sensor and sizes of the sensor can be redesigned according to the critical crack length of the structure under monitoring. Because eddy current testing is a non-contact detection method, the sensor can be directly mounted on critical components of the structure using sealants without any other surface treatment. What operators need to do is to make sure the sense channels cover the welding seam where cracks are most likely to initiate and grow.

## 6. Conclusions

This paper mainly investigates FECA sensors for crack monitoring of welded structures under stress and temperature interference.

Firstly, experimental research on effects of stress, temperature and crack on the output signal of the traditional sensor was carried out. Experimental results show that the sensitivity of the sensor to cracks is so low that stress and temperature variations have great influences on the crack monitoring process. Then, a 3-D FE model was established to analyze the perturbation of the crack to eddy currents. According to results, the reason the sensitivity of the sensor is so low is because adjacent exciting currents in the opposite direction forms current loops when the crack propagates, making eddy currents at the crack tip decrease, reducing the perturbation effect of the crack on eddy currents.

Therefore, the HS-FECA sensor with a new winding method in which the exciting currents are arranged in the same direction was proposed. FE simulation reveals that the new winding method makes the perturbation effect of the crack on eddy currents stronger and stronger when the crack propagates, resulting in much higher sensitivity to cracks. The following experiments further prove that the sensitivity of the new sensor is at least 19 times that of the original one, both stress and temperature variations have little effects on output signals of the new sensor.

## References

[1] Smith T.A., Warwick R.G. (1974). A survey of defects in pressure vessels built to high standards of construction and its relevance to nuclear primary circuits. Int. J. Press. Vessels Pip..

[2] Zhao Z. (2001). Research and Application of Crack Diagnosis, Control and Maintenance Method for Mechanical Bearing Structures.

[3] Majumder M., Gangopadhyay T.K., Chakraborty A.K., Dasgupta K., Bhattacharya D.K. (2008). Fibre Bragg gratings in structural health monitoring—Present status and applications. Sens. Actuators A Phys..

[4] Kinet D., Mégret P., Goossen K.W., Qiu L., Heider D., Caucheteur C. (2014). Fiber Bragg grating sensors toward structural health monitoring in composite materials: Challenges and solutions. Sensors.

[5] Rodríguez G., Casas J R., Villaba S. (2015). Cracking assessment in concrete structures by distributed optical fiber. Smart Mater. Struct..

[6] Oromiehie E., Prusty B.G., Compston P., Rajan G. (2018). Characterization of process-induced defects in automated fiber placement manufacturing of composites using fiber Bragg grating sensors. Struct. Health Monit..

[7] Stehmeier H., Speckmann H. Comparative Vacuum Monitoring (CVM) Monitoring of fatigue cracking in aircraft structures. Proceedings of the 2nd European Workshop on Structural Health Monitoring.

[8] Racle E., Godin N., Reynaud P., Fantozzi G. (2017). Fatigue Lifetime of Ceramic Matrix Composites at Intermediate Temperature by Acoustic Emission. Materials.

[9] Farnam Y., Geiker M.R., Bentz D., Weiss J. (2015). Acoustic emission waveform characterization of crack origin and mode in fractured and ASR damaged concrete. Cem. Concr. Compos..

[10] Cho H., Lissenden C.J. (2012). Structural health monitoring of fatigue crack growth in plate structures with ultrasonic guided waves. Struct. Health Monit..

[11] Smithard J., Rajic N., van der Velden S., Norman P., Rosalie C., Galea S., Mei H., Lin B., Giurgiutiu V. (2017). An Advanced Multi-Sensor Acousto-Ultrasonic Structural Health Monitoring System: Development and Aerospace Demonstration. Materials.

[12] Liu Z., Chen K., Li Z., Jiang X. (2017). Crack Monitoring Method for an FRP-Strengthened Steel Structure Based on an Antenna Sensor. Sensors.

[13] Roy S., Lonkar K., Janapati V., Chang F.K. (2014). A novel physics-based temperature compensation model for structural health monitoring using ultrasonic guided waves. Struct. Health Monit..

[14] Rakow A., Chang F.K. (2012). A structural health monitoring fastener for tracking fatigue crack growth in bolted metallic joints. Struct. Health Monit..

[15] Goldfine N., Grundy D., Craven C., Washabaugh A., Zilberstein V., Sheiretov Y., Weiss V., Davis M., Schaff J., Hullander T. Damage and usage monitoring for vertical flight vehicles. Proceedings of the Annual Forum Proceedings—American Helicopter Society.

[16] Sheiretov Y., Grundy D., Zilberstein V., Goldfine N., Maley S. (2009). MWM-array sensors for in situ monitoring of high-temperature components in power plants. IEEE Sens. J..

[17] Goldfine N., Denenberg S., Manning B., Thomas Z., Al Rushaid R., Haught F. Modeling and Visualization for Imaging of Subsurface Damage. Proceedings of the 7th Middle East NDT Conference & Exhibition.

[18] Goldfine N., Manning B., Thomas Z., Sheiretov Y., Denenberg S., Dunford T., Haque S., Al Rushaid R., Haught F. Imaging Corrosion under Insulation and under Fireproofing, using MR-MWM-Arrays. Proceedings of the 7th Middle East NDT Conference & Exhibition.

[19] Neil G., David G., Jennifer M., Manning B., Martin C., Root C., Nguyen A., Engstrand C., Kulowitch P., Barrett A. Flight Testing of Permanently Installed Eddy Current Sensors for IVHM. Proceedings of the 2014 Aircraft Airworthiness and Sustainment Conference.

[20] He Y., Chen T., Du J., Ding H., Jiao S., Li P. (2017). Temperature-compensated rosette eddy current array sensor (TC-RECA) using a novel temperature compensation method for quantitative monitoring crack in aluminum alloys. Smart Mater. Struct..

[21] (2015). MULTIPHYSICS, COMSOL. v. 5.2.

